# Housing Insecurity, Behavioral Health Disorders, and Acute Care Utilization: A Cross-Sectional Study of Medicaid Members in the Portland, Oregon Metropolitan Area

**DOI:** 10.1007/s11524-026-01073-3

**Published:** 2026-04-27

**Authors:** Megan Holtorf, Daniel J. Fish, Kristen A. Lacijan, Benjamin Gronowski, Graham Bouldin, Andrew Mendenhall, Taylor Doren, Hannah Cohen-Cline, Catherine J. Livingston

**Affiliations:** 1 Center for Outcomes Research and Education (CORE), Providence, Portland, OR USA; 2 Health Share of Oregon, Portland, OR USA

**Keywords:** Substance use disorder, Opioid use disorder, Medications for opioid use disorder, Housing instability, Acute care utilization

## Abstract

Housing insecurity and substance use disorder (SUD) are critical public health issues in the USA, with significant implications for health outcomes. This study evaluated the intersection of housing insecurity, SUD, and psychosis among Medicaid enrollees in an urban center in Oregon. Using data from Health Share of Oregon, we identified three distinct cohorts—individuals with housing insecurity, those with SUD/psychosis, and those with both conditions. Key outcomes included inpatient admission rates, average lengths of stay, and 30-day readmissions. The findings indicate that housing-insecure individuals with SUD/psychosis show greater acute care utilization than the other two cohorts. Inpatient admissions for housing-insecure individuals with SUD/psychosis were over twice as high as those without housing insecurity (29.7% vs. 12.4%), nearly three times as high as those without SUD/psychosis (29.7% vs. 9.5%), and almost ten times higher than those without either (29.7% vs. 3.0%). There were likewise corresponding increases in ambulatory-sensitive hospitalizations, 30-day readmissions, and longer hospital stays. Effective interventions should address both housing and healthcare needs, including comprehensive case management and improved access to physical, behavioral, and mental health services integrated with housing programs and services. These strategies could mitigate acute care utilization and improve overall health outcomes for these vulnerable populations.

## Introduction

Homelessness is an increasing public health concern across the USA, and per the US Department of Housing and Urban Development is defined as lacking a fixed, regular, and adequate nighttime residence [1]. In 2023, the US Annual Homelessness Assessment Report (AHAR)’s Point-In-Time count recorded the highest number of people experiencing homelessness on a single night since reporting began in 2007; according to the National Alliance to End Homelessness (NAEH), the majority were concentrated in urban areas [2, 3]. The number of people in 2023 experiencing homelessness was approximately 653,100, representing a 12% increase between 2020 and 2023 [2].

The state of Oregon, particularly the Portland, Oregon metro area, is experiencing one of the worst housing crises, with this region having among the highest per-capita rate of unsheltered homelessness in the USA [4]. The point in time estimates for the tricounty area (Clackamas, Multnomah and Washington counties) report that 12,034 people—or roughly 66 per 10,000—were experiencing homelessness in a single night in 2025; a rate substantially higher than the national homelessness rate of 23 per 10,000 people [5–7]. There is therefore a pressing need for greater access to shelters and diverse types of housing, including respite, transitional, and permanent housing. This is particularly true in the Portland Metro area, which has roughly 75% of the state’s homeless population while making up only 43% of the state’s total population [6, 8–11].

Homelessness has a profound impact on health: a literature review of 42 studies found that people experiencing unsheltered homelessness have higher rates of mortality, chronic diseases, serious mental illness (SMI), and substance use compared to sheltered populations [2, 12]. Substance use disorders (SUD) and SMI are particularly associated with housing instability and can lead to worse health for this population; Stablein et al. found a bidirectional relationship between SUD diagnoses and homelessness, and that homelessness was associated with more severe SUD, greater risk of medical comorbidities, and poorer health outcomes [13]. Ayangbayi et al. found that people experiencing homelessness are more than six times more likely to have an Emergency Department (ED) visit for mental health and SUD concerns [14].

Both housing insecurity and SUD are disproportionately represented in the Medicaid population, necessitating strategies specifically addressing this population [15]. Given the intersections between SUD, serious mental illness, homelessness, and associated morbidities, understanding the acute care utilization patterns across people with housing insecurity and SUD (and those with both) is essential for developing interventions. 

While it is generally understood from the literature that individuals with SUD, SMI, or housing insecurity have higher morbidity and mortality rates than the general population, there are fewer studies that focus specifically on the impact of housing instability as a factor associated with acute care utilization within this population. This study is a collaborative effort across regional organizations with the aim of developing and implementing care coordination, clinical, and payment model interventions for Medicaid populations.

Oregon’s largest coordination care organization, Health Share of Oregon, which serves approximately 450,000 Oregon Health Plan (Medicaid) members, is working with regional health care systems and behavioral health organizations to develop an approach to understand the intersections between SUD, SMI, housing insecurity, and acute care utilization, with the aim of ultimately improving outcomes for Medicaid members. 

This study aims to enhance the understanding of the intersection of housing instability, SUD, and SMIs, with the ultimate goal of implementing policy decisions and resource allocation which will prevent avoidable morbidity and mortality. Medicaid is the single largest behavioral health services payer in the USA, and its interventions and initiatives can reach a significant number of people.

## Methods

### Data Sources

We conducted a cross-sectional analysis utilizing Medicaid enrollment and claims data from Health Share of Oregon, the largest coordinated care organization (CCO) in Oregon. Health Share of Oregon serves approximately 450,000 members across three counties in the Portland metro area. The data included information on member demographics, diagnosis codes, healthcare utilization, and participation in specific housing support programs.

### Study Population

The study sample comprised adult Health Share members aged 18 and older who were enrolled for a minimum of 6 months during the calendar year 2023. The study’s advisory group, which was comprised of staff at Health Share, CareOregon (an integrated community network health plan managing specialty behavioral, physical, and oral health services), Central City Concern (a regional integrated housing and health care Federally Qualified Health Center, regional behavioral health leaders, and a community-based peer organization including those with lived experience, provided direction on priority areas of data analysis. Specifically, the advisory group recommended focusing on a high acuity cohort, including opioid use disorder, stimulant use disorder, psychosis, and unintentional substance-associated overdose. While broader SMI conditions were considered, the group recommended focusing on psychosis as the critical mental health concern due to long-term regional inadequate inpatient psychiatric capacity (a primary reason for needing acute inpatient psychiatric care) and the active harms experienced in supportive housing, health care organizations, and community by unmet need for acute psychosis treatment (e.g., interpersonal violence occurring in housing units and behavioral health facilities, and arson, among others).

We identified individuals with substance use disorder (SUD) and Psychotic Disorders through ICD-10-CM diagnosis codes in Medicaid claims data. Individuals were identified as housing insecure if they had a claim during 2023 with a diagnosis of homelessness (Z59.0), inadequate housing (Z59.1), housing instability (Z59.81), or other problems related to housing and economic circumstances (Z59.89); if they were identified as homeless in the state’s Medicaid 834 benefit enrollment and maintenance file; or if they participated in Health Share’s housing benefit pilot, a program that provided temporary rental assistance, housing navigation, and related housing supports to Medicaid members at risk of homelessness [16].

Diagnosis codes for SUD and Psychotic Disorders were developed collaboratively by Health Share, CareOregon, and Central City Concern. Individuals dually enrolled in Medicaid and Medicare were excluded during months of dual coverage. To reduce potential misclassification, individuals with no medical claims for 2023 were excluded from the analysis.

### Measures

Key demographic and social variables included age, race/ethnicity, preferred language, disability status, gender, and housing insecurity status. Healthcare utilization measures included the percentage of individuals with at least one emergency department (ED) or primary care provider (PCP) visit, and the frequency of these visits per 1000 member months during calendar year 2023.

Primary outcomes included inpatient medical admissions, hospitalizations for ambulatory-sensitive conditions, 30-day all-cause readmissions, and average length of stay (ALOS), all measured during the calendar year 2023. Inpatient admissions related to maternity were excluded. Readmission rates were calculated by identifying hospitalizations within 30 days of discharge, with only the first readmission counted and transfers combined into a single record. ALOS was calculated as the mean number of days between admission and discharge for each unique hospitalization, with transfers combined. ALOS was calculated only for those who were hospitalized for one or more days. Hospitalizations for ambulatory-sensitive conditions were identified using standard definitions, and primary diagnoses for inpatient admissions were categorized using the AHRQ Clinical Classifications Software [17].

### Statistical Analysis

Descriptive statistics summarized population characteristics and healthcare utilization across the cohorts. For continuous variables including age and length of stay, means and standard deviations were reported. Frequencies and percentages were calculated for categorical variables such as race/ethnicity and gender. Rates for healthcare utilization measures, including ambulatory-sensitive hospitalizations, inpatient admissions, 30-day all-cause readmissions, and length of stay, were calculated per 1000 member months as well as frequencies and percentages.

We fit binary healthcare utilization outcomes with a modified Poisson distribution with a log link. For utilization per 1000 member months, we fit a generalized estimating equation with a Gaussian distribution. Lastly, we fit length of stay outcomes with a Poisson distribution. After fitting the models, we estimated the average marginal effects using the marginaleffects package in R [18].

Individuals with any combination of housing insecurity or SUD/Psychotic Disorders were compared to those with neither. All models were adjusted for age (continuous) at the end of 2023, language, gender, and presence of any chronic condition diagnosis on a claim during 2023. All statistical analyses were performed using R software (version 4.3.1).

### Ethical Considerations

This study adhered to ethical guidelines. The study protocol was reviewed and approved by the Providence Health and Services Institutional Review Board (IRB); the IRB granted a waiver of informed consent.

## Results

### Population Characteristics

Table 1 describes population characteristics by housing insecurity status and SUD/Psychotic Disorder diagnosis. There were 19,986 enrollees with and 160,077 enrollees without a SUD/Psychotic Disorder. Of those without a SUD/Psychotic Disorder diagnosis, approximately 3% were identified as housing insecure; of those with a SUD/Psychotic Disorder diagnosis, approximately 24% were identified as housing insecure. In both groups, housing insecure individuals were slightly older, more likely to identify as Black/African American, and less likely to identify as American Indian/Alaska Native or Asian compared to housing-secure individuals. Among those with a SUD/Psychotic Disorder diagnosis, housing insecure enrollees were also more likely to be male compared to housing-secure individuals.

**Table 1:** Population characteristics by housing insecurity status and SUD/Psychotic Disorder diagnosis, (2023) (source/notes: authors’ analysis of Medicaid claims and enrollment data)

	No. SUD/Psychotic Disorder, *N* = 160,077			Any SUD/Psychotic Disorder, *N* = 19,986		
	Overall *N* = 160,077^1^	Not housing insecure *N* = 154,913^1^	Housing insecure *N* = 5164^1^	Overall *N* = 19,986^1^	Not housing insecure *N* = 15,213^1^	Housing insecure *N* = 4773^1^
Age	38.28 (14.00)	38.11 (13.99)	43.35 (13.34)	41.69 (11.90)	41.44 (12.04)	42.47 (11.44)
Race and ethnicity						
Asian	13,807 (8.6%)	13,555 (8.8%)	252 (4.9%)	814 (4.1%)	687 (4.5%)	127 (2.7%)
Black or African American	15,224 (9.5%)	14,444 (9.3%)	780 (15.1%)	2028 (10.1%)	1475 (9.7%)	553 (11.6%)
White	70,319 (43.9%)	67,693 (43.7%)	2626 (50.9%)	11,916 (59.6%)	9017 (59.3%)	2899 (60.7%)
American Indian/Alaska Native	17,581 (11.0%)	17,071 (11.0%)	510 (9.9%)	2139 (10.7%)	1697 (11.2%)	442 (9.3%)
Latino/a/x/e	28,594 (17.9%)	28,011 (18.1%)	583 (11.3%)	1596 (8.0%)	1246 (8.2%)	350 (7.3%)
Native Hawaiian/Pacific Islander	2363 (1.5%)	2285 (1.5%)	78 (1.5%)	230 (1.2%)	172 (1.1%)	58 (1.2%)
Middle Eastern/North African	3098 (1.9%)	3024 (2.0%)	74 (1.4%)	169 (0.8%)	137 (0.9%)	32 (0.7%)
Multiple or not listed	3142 (2.0%)	3018 (1.9%)	124 (2.4%)	407 (2.0%)	324 (2.1%)	83 (1.7%)
Unknown	5949 (3.7%)	5812 (3.8%)	137 (2.7%)	687 (3.4%)	458 (3.0%)	229 (4.8%)
Language						
English	115,281 (72.0%)	110,889 (71.6%)	4392 (85.1%)	17,752 (88.8%)	13,471 (88.5%)	4281 (89.7%)
Spanish	16,349 (10.2%)	16,104 (10.4%)	245 (4.7%)	321 (1.6%)	263 (1.7%)	58 (1.2%)
Other	28,447 (17.8%)	27,920 (18.0%)	527 (10.2%)	1913 (9.6%)	1479 (9.7%)	434 (9.1%)
Gender						
Female	95,044 (59.4%)	92,078 (59.4%)	2966 (57.4%)	9493 (47.5%)	7499 (49.3%)	1994 (41.8%)
Male	65,033 (40.6%)	62,835 (40.6%)	2198 (42.6%)	10,493 (52.5%)	7714 (50.7%)	2779 (58.2%)
Chronic conditions						
None	37,453 (23.7%)	37,235 (24.3%)	218 (4.7%)	218 (0.8%)	192 (0.9%)	26 (0.4%)
One or more	120,312 (76.3%)	115,918 (75.7%)	4394 (95.3%)	25,966 (99.2%)	20,035 (99.1%)	5931 (99.6%)
Emergency department visit						
Members with at least one ED visit	45,749 (28.6%)	43,046 (27.8%)	2703 (52.3%)	11,215 (56.1%)	7546 (49.6%)	3669 (76.9%)
Utilization per 1000 mm	42.08 (98.59)	39.61 (90.43)	116.15 (224.25)	155.11 (396.83)	101.03 (188.98)	327.47 (711.77)
Primary care visits						
Members with at least one primary care visit	125,014 (78.1%)	120,688 (77.9%)	4326 (83.8%)	17,112 (85.6%)	13,351 (87.8%)	3761 (78.8%)
Utilization per 1000 mm	248.12 (328.99)	243.23 (322.71)	394.71 (457.08)	466.22 (553.77)	461.34 (521.83)	481.80 (644.91)

^1^Mean (SD); *n* (%)

*mm* member months

In both groups with and without a SUD/Psychotic Disorder diagnosis, Emergency department (ED) visits were substantially higher among the housing insecure group, both in terms of the percent of individuals having at least one ED visit and the total number of ED visits per 1000 member months. In contrast, there were smaller and less consistent differences in primary care visits; among those with a SUD/Psychotic Disorder diagnosis, housing insecure enrollees were less likely to have a primary care visit, but the frequency of primary care visits per 1000 member months was higher compared to the housing-secure group.

### Acute Care Utilization

Table 2 presents acute care utilization rates, revealing large differences by housing insecurity status. Inpatient admission rates were significantly higher among individuals with housing insecurity (9.5% vs. 3.0% for those without SUD/Psychotic Disorder, and 29.7% vs. 12.4% for those with SUD/Psychotic Disorder). Utilization rates per 1000 member months were also notably higher among those with SUD/Psychotic Disorder, with housing insecure individuals experiencing 52.53 inpatient admissions per 1000 member months compared to 17.23 in their housing-secure counterparts.

**Table 2:** Acute care utilization by housing insecurity status and SUD/Psychotic Disorder diagnosis, (2023) (source/notes: authors’ analysis of Medicaid claims data)

Characteristic	No SUD/Psychotic Disorder, *N* = 160,077			Any SUD/Psychotic Disorder, *N* = 19,986		
	Overall *N* = 160,077^1^	Not housing insecure *N* = 154,913^1^	Housing insecure *N* = 5164^1^	Overall *N* = 19,986^1^	Not housing insecure *N* = 15,213^1^	Housing insecure *N* = 4773^1^
Inpatient admission						
Any utilization	5127 (3.2%)	4635 (3.0%)	492 (9.5%)	3298 (16.5%)	1882 (12.4%)	1416 (29.7%)
Utilization per 1000 mm	4.13 (29.01)	3.82 (27.71)	13.66 (54.46)	25.66 (82.36)	17.23 (60.21)	52.53 (126.10)
Hospitalization for an ambulatory sensitive condition (ASC)						
Members with at least one hospitalization for ASC	461 (0.3%)	405 (0.3%)	56 (1.1%)	395 (2.0%)	223 (1.5%)	172 (3.6%)
Utilization per 1000 mm	0.32 (7.40)	0.29 (6.94)	1.35 (15.96)	2.55 (26.10)	1.68 (17.20)	5.30 (43.58)
Inpatient readmission in 30 days						
Members with at least one readmission	900 (0.6%)	802 (0.5%)	98 (1.9%)	804 (4.0%)	403 (2.6%)	401 (8.4%)
Utilization per 1000 mm	0.78 (13.60)	0.73 (13.30)	2.40 (20.69)	6.67 (48.92)	3.71 (30.06)	16.10 (83.82)
Inpatient days per 1000 mm	0.95 (10.64)	0.87 (10.24)	3.48 (18.86)	6.88 (31.19)	4.54 (25.31)	14.34 (44.26)
Length of stay	6.91 (9.45)	6.82 (9.49)	7.73 (9.12)	8.15 (12.10)	7.97 (12.37)	8.35 (11.80)

^1^*n* (%); mean (SD)

*mm* member months

Hospitalizations for ambulatory-sensitive conditions and 30-day inpatient readmissions were also disproportionately higher among housing insecure individuals with SUD/Psychotic Disorder. For example, 3.6% of housing insecure individuals with SUD/Psychotic Disorder had ambulatory-sensitive hospitalizations, compared to 1.5% of housing-secure individuals. Similarly, the 30-day inpatient readmission rate was 8.4% for housing insecure individuals with SUD/Psychotic Disorder, compared to 2.6% for their housing-secure counterparts.

### Effect of Housing Insecurity and Cohort Status on Acute Care Utilization

Table 3 illustrates the compounded effect of housing insecurity and SUD/Psychotic Disorder on acute care utilization. The housing insecure and SUD/Psychotic Disorder group exhibited the largest differences in utilization rates. Housing insecure individuals with SUD/Psychotic Disorder had an 18.3 percentage point increase (95% CI, 17.2%, 19.5%; *p* < 0.001) for inpatient admissions, with an increase of 46.6 hospitalizations per 1000 member months (95% CI, 45.5, 47.7).

**Table 3:** Average absolute differences in acute care utilization associated with housing insecurity and presence of SUD/psychosis disorder, compared to housing-secure individuals without a SUD/psychosis disorder diagnosis, (2023) (source/notes: authors’ analysis of Medicaid claims data)

Outcome	Any utilization				Total utilization			
	Unadjusted AME	95% CI	Adjusted AME^1^	95% CI^1^	Unadjusted AME	95% CI	Adjusted AME^1^	95% CI^1^
Inpatient admissions								
Not housing insecure and no SUD/Psychotic Disorder	*ref*	—	*ref*	—	*ref*	—	*ref*	—
Housing insecure and no SUD/Psychotic Disorder	6.50%	5.7%, 7.4%**	4.10%	3.5%, 4.8%**	9.84	8.78, 10.9**	8.05	6.99, 9.12**
Not housing insecure and any SUD/Psychotic Disorder	9.40%	8.8%, 9.9%**	6.30%	5.8%, 6.7%**	13.4	12.8, 14.0**	11.7	11.0, 12.3**
Housing insecure and any SUD/Psychotic Disorder	26.70%	25.1%, 28.2%**	18.30%	17.2%, 19.5%**	48.7	47.6, 49.8**	46.6	45.5, 47.7**
Hospitalizations for an ambulatory sensitive condition								
Not housing insecure and no SUD/Psychotic Disorder	*ref*	—	*ref*	—	*ref*	—	*ref*	—
Housing insecure and no SUD/Psychotic Disorder	0.80%	0.5%, 1.1%**	0.50%	0.3%, 0.7%**	1.05	0.746, 1.36**	0.904	0.594, 1.21**
Not housing insecure and any SUD/Psychotic Disorder	1.20%	1.0%, 1.4%**	0.80%	0.7%, 1.0%**	1.39	1.21, 1.58**	1.25	1.06, 1.44**
Housing insecure and any SUD/Psychotic Disorder	3.30%	2.8%, 3.9%**	2.30%	1.9%, 2.7%**	5.01	4.69, 5.33**	4.83	4.51, 5.16**
Inpatient readmission in 30 days								
Not housing insecure and no SUD/Psychotic Disorder	*ref*	—	*ref*	—	*ref*	—	*ref*	—
Housing insecure and no SUD/Psychotic Disorder	1.40%	1.0%, 1.8%**	0.90%	0.6%, 1.1%**	1.68	1.10, 2.25**	1.28	0.707, 1.86**
Not housing insecure and any SUD/Psychotic Disorder	2.10%	1.9%, 2.4%**	1.50%	1.3%, 1.7%**	2.99	2.65, 3.33**	2.65	2.30, 3.00**
Housing insecure and any SUD/Psychotic Disorder	7.90%	7.1%, 8.7%**	5.50%	4.9%, 6.2%**	15.4	14.8, 16.0**	14.9	14.3, 15.5**
Inpatient days								
Not housing insecure and no SUD/Psychotic Disorder					*ref*	—	*ref*	—
Housing insecure and no SUD/Psychotic Disorder					2.61	2.22, 3.01**	2.15	1.75, 2.55**
Not housing insecure and any SUD/Psychotic Disorder					3.67	3.43, 3.91**	3.26	3.02, 3.50**
Housing insecure and any SUD/Psychotic Disorder					13.5	13.1, 13.9**	12.9	12.5, 13.4**
Length of stay								
Not housing insecure and no SUD/Psychotic Disorder					*ref*	—	*ref*	—
Housing insecure and no SUD/Psychotic Disorder					0.91	0.116, 1.70*	0.743	0.541, 0.945**
Not housing insecure and any SUD/Psychotic Disorder					1.15	0.691, 1.62**	1.07	0.951, 1.19**
Housing insecure and any SUD/Psychotic Disorder					1.53	1.06, 2.00**	1.54	1.42, 1.67**

*AME* average marginal effect, *CI* confidence interval

^1^Adjusted for age (continuous), language, gender, and presence of any chronic conditions

*p < 0.05, **p < 0.001

For hospitalizations due to ambulatory-sensitive conditions, the percentage point increase for housing insecure individuals with SUD/Psychotic Disorder was 2.3% (95% CI, 1.9%, 2.7%; *p* < 0.001), and an increase of 4.83 hospitalizations per 1000 member months (95% CI, 4.51, 5.16) when compared to individuals without housing insecurity or SUD/Psychotic Disorder. Similarly, 30-day inpatient readmissions were substantially elevated in this group, with percentage point increase of 5.5% (95% CI, 4.9%, 6.2%; *p* < 0.001), and an increase of 14.9 readmissions per 1000 member months (95% CI, 12.5, 13.4).

Length of stay also increased with housing insecurity and SUD/Psychotic Disorder diagnosis. Housing insecure individuals with SUD/Psychotic Disorder had an average length of stay 1.54 days longer than those without housing insecurity or SUD/Psychotic Disorder (95% CI, 0.951, 1.19; *p* < 0.001).

### Sensitivity Analyses

Approximately 28% of the eligible population had no claims in calendar year 2023. Individuals with and without claims were similar on most demographic characteristics (data not shown); however, those with claims were more likely to be female (58% vs 42%) and to indicate English as their preferred language (76% vs 68%). When individuals without claims were included in the analysis, the results did not substantially change from the main analysis (data not shown).

## Discussion

This study represents a collaborative partnership across regional organizations serving Medicaid individuals in the Portland metro area with the goal of driving clinical, care coordination, health-related social needs, and payment model interventions. The findings show that housing insecure individuals with co-occurring SUD/psychosis have significantly higher acute care use compared to individuals without housing insecurity or without SUD/psychosis. Further, experiencing both housing insecurity and SUD/psychosis had a compound effect on acute care utilization, with much higher inpatient utilization, longer length of stays, and more frequent 30-day readmissions among this group compared to those experiencing just housing instability or just SUD/psychosis. This suggests that greater housing stability could reduce acute care needs in a population with complex behavioral health needs.

Several factors may contribute to higher acute care use and longer length of stays in this population, including more severe and chronic comorbid conditions, inability to effectively manage SUD during hospitalization with models such as Addiction Consult Services, and difficulties safely discharging patients to a suitable environment to recover from severe illness or active SUD, which prolongs hospital stays and increases readmission risk [19]. For example, it can be difficult for housing insecure individuals to effectively manage chronic leg wounds (both in terms of wound care and taking antibiotics), resulting in recurrent cellulitis and necessitating follow up ED visits and readmissions [2, 8–11].

There are potential opportunities to improve morbidity in this population. For people with SUD in acute care settings, ensuring they have access to evidence-based MOUD care is critical and facilitates improvement of comorbid medical conditions, and effective linkage to outpatient SUD care. Evidence suggests that models such as IMPACT and increasing Emergency Department MOUD prescribing and linkage to care, and expanding access to contingency management for stimulant use disorder can improve engagement and reduce mortality rates [19–22].

Alongside clinical system-based strategies, improving housing stability through the housing continuum for people with SUD and psychosis is important. This can involve newly aligned strategies between the health care and housing service continuum, such as improving access to 24/7 shelters with onsite health supports and post-hospitalization medical respite. These services provide a conducive environment for recovery, better sanitary and physical conditions for wound care and healing, and support engagement with service and treatment providers. Post-hospitalization medical respite can effectively reduce readmission rates among individuals who are unhoused [23]. Additionally, implementing low barrier (non-alcohol and drug free) permanent housing programs can help mitigate some comorbid factors due to improved sanitary and environmental conditions. Transitional recovery housing programs linked to case management and care coordination can support individuals seeking treatment and recovery pathways, contributing to long-term stability [24]. Finally, at a national level, funding could be integrated from the Department of Housing and Urban Development (HUD) and the Centers for Medicare and Medicaid Services (CMS), ensuring that both rent and integrated health services are adequately funded.

### Limitations

The results of this study should be interpreted within the context of its limitations. First, the study’s reliance on diagnosis codes means undiagnosed conditions may be misclassified, potentially underestimating the affected population. The housing instability flag likely underestimates housing insecurity due to variable coding practices among providers [25] and reflects housing insecurity identified through the health care system alone, rather than also (or alternatively) through the housing sector. 

Second, we focused on psychotic disorders instead of broader SMI conditions. While this decision was driven by recognition of specific regional needs around psychosis, it does mean the results may not be generalizable to populations with other SMIs. 

Additionally, our analysis excluded individuals with no utilization who comprised approximately 28% of the eligible population. While our sensitivity analysis found no qualitative differences when including these individuals, this represents a large proportion of the population who are not engaging with the healthcare system and for whom our results may not be fully generalizable. Finally, our results also may not be completely generalizable to other urban populations with a different distribution of demographic characteristics, different access to housing resources, or different health care systems.

### Suggestions for Future Research

Building on these findings, future research should investigate how specific types of housing instability affect health outcomes and healthcare utilization among individuals with SUD and SMI. It is crucial to understand how different kinds of housing challenges, whether temporary or chronic, uniquely impact the well-being and medical needs of these populations. Leveraging data from the housing sector, via the Homeless Management Information System, would further enable more extensive data analysis about those people interfacing with specific housing sector programs interlaced with the housing claims data. Similarly, studies focusing on what housing, care coordination, and clinical strategies improve outcomes for this population would be valuable. These insights could help inform strategies designed to improve service access, engagement, and clinical outcomes across health care and housing programs.

Examining patterns among subgroups and identifying factors that contribute to longer hospital stays—such as patient acuity or available discharge options—could provide important insights for tailoring interventions more effectively. Additionally, understanding the temporal relationship between the onset of housing instability and the escalation in healthcare utilization could inform early intervention models. These models would be crucial for mitigating adverse health impacts and reducing unnecessary hospital admissions, ultimately leading to more effective and personalized care strategies for those affected by SUD and SMI.

## Conclusions

This study underscores the critical impact of housing instability on acute care utilization among individuals with SUD and psychosis within the Medicaid population in Oregon. The findings demonstrate that housing insecure individuals exhibit markedly higher rates of inpatient admissions, longer lengths of stay, and increased 30-day readmission rates compared to their housing-secure counterparts. These patterns highlight the pressing need for policy interventions that integrate housing support with health care services to reduce acute care use. Strategies such as providing low-barrier access to medication for opioid use disorder, enhancing shelter and respite services, improving coordination between health services and housing programs, and leveraging HUD and CMS funding streams could be instrumental in addressing the complex needs of these vulnerable populations. Addressing housing instability as a health-related social need is pivotal to improving outcomes for Medicaid beneficiaries with intersecting behavioral health conditions and housing challenges.

## Data Availability

The Medicaid enrollment and claims data used in this study is not publicly available
